# Supplementary material for: Determination of Mercury in Fish Sauces by Thermal Decomposition Gold Amalgamation Atomic Absorption Spectroscopy after Preconcentration by Diffusive Gradients in Thin Films Technique
Source: Foods. 2020 Dec 12;9(12):1858. doi: 10.3390/foods9121858 (PMC7764611; doi:10.3390/foods9121858)
Supplement: Supplementary file 1 [file foods-09-01858-s001.pdf]

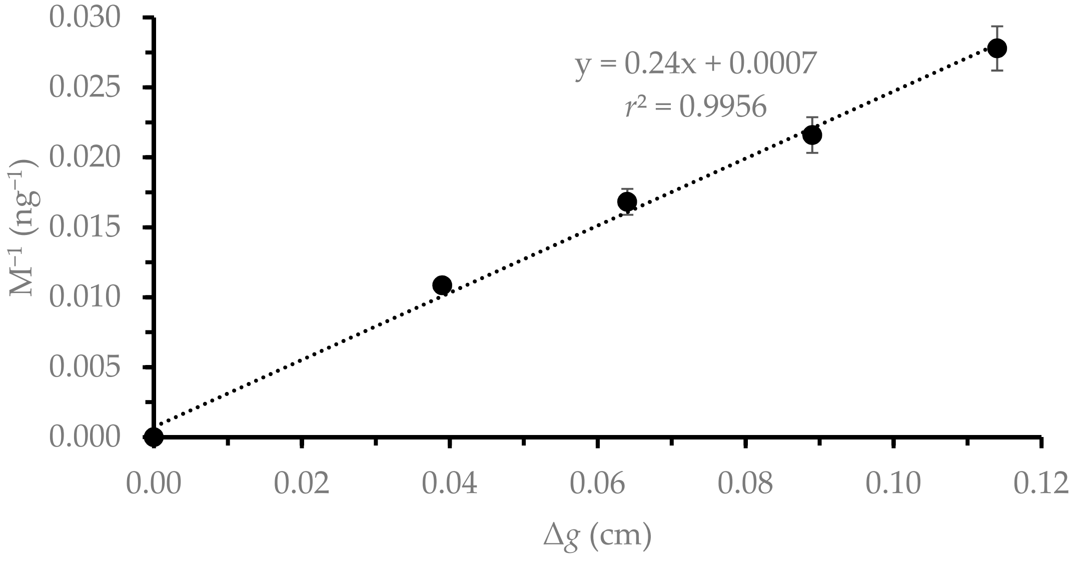

Figure S1. Measured mass of mercury in the binding gels immersed in Hg model solution for different thicknesses of diffusive gel.
